# DNA methylation signatures of incident coronary heart disease: findings from epigenome-wide association studies

**DOI:** 10.1186/s13148-021-01175-6

**Published:** 2021-10-09

**Authors:** Yujing Xia, Alison Brewer, Jordana T. Bell

**Affiliations:** 1 Department of Twin Research and Genetic Epidemiology, Kings College London, London, SE1 7EH UK; 2 School of Cardiovascular Medicine and Sciences, James Black Centre, King’s College London British Heart Foundation Centre of Excellence, 125 Coldharbour Lane, London, SE5 9NU UK

**Keywords:** DNA methylation, Incident coronary heart disease, Epigenome-wide association study

## Abstract

Coronary heart disease (CHD) is a type of cardiovascular disease (CVD) that affects the coronary arteries, which provide oxygenated blood to the heart. It is a major cause of mortality worldwide. Various prediction methods have been developed to assess the likelihood of developing CHD, including those based on clinical features and genetic variation. Recent epigenome-wide studies have identified DNA methylation signatures associated with the development of CHD, indicating that DNA methylation may play a role in predicting future CHD. This narrative review summarises recent findings from DNA methylation studies of incident CHD (iCHD) events from epigenome-wide association studies (EWASs). The results suggest that DNA methylation signatures may identify new mechanisms involved in CHD progression and could prove a useful adjunct for the prediction of future CHD.

## Background

Coronary heart disease (CHD) is the most common cardiovascular disease (CVD) and a leading cause of death worldwide, with an estimated 8.88 million deaths from CHD in 2019 globally [1]. Atherosclerosis, a long-term inflammatory process in the arteries, is the common basis for CHD [2]. Atherosclerosis leads to the build-up of atherosclerotic plaques, and involves lipid deposition, inflammatory cells migration, smooth muscle cell proliferation and fibrosis. Eventually, the formation of an advanced atherosclerotic plaque narrows the artery, causing blood clots at the site of the lesion. Alternatively, the plaque may rupture and lead to blood clots in downstream arteries. CHD takes place when blood flow in coronary arteries is reduced or blocked due to the impact of atherosclerosis. The outcomes of CHD can be classified according to the severity of blockage of the vessel, from mild outcomes such as stable angina to severe acute coronary syndrome. The former is caused by a temporary insufficiency of blood flow supplying the heart during physical activity or stress. The latter refers to a sudden occurrence of more severe forms of coronary heart events which happen at rest, for example, in unstable angina and in myocardial infarction (MI), caused by complete blockage of coronary arteries.

The development of CHD is a long-term process, and certain risk factors can accelerate or worsen coronary atheroma development and the chance of a severe CHD event. Risk factors considered here include lifestyle factors and phenotypes that have a causal relationship with the increased risk of developing CHD. Acknowledged risk factors for CHD include smoking, hypertension, type 2 diabetes, and hyperlipidaemia, which corresponds to elevated levels of triglycerides (TG) and low-density lipoprotein (LDL) cholesterol, or low levels of high-density lipoprotein (HDL) cholesterol. Other risk factors also include high levels of C-reactive protein (CRP), lipoprotein-A, haemoglobin A1c (HbA1c), obesity, family history of CHD, and others [3]. Assessment of risk factors can be used to identify asymptomatic individuals at increased risk of CHD, especially in primary health care settings, and this in turn can lead to different levels of interventions. For low- and moderate-risk individuals, interventions are normally lifestyle modifications, while for high-risk individuals pharmacological intervention may be necessary. To reduce the risk of severe outcomes, individuals with high cardiovascular risk, or mild CHD symptoms, or high-risk coronary atherosclerosis conditions diagnosed by direct plaque imaging often receive medical treatment to slow down the formation of atherosclerotic plaques. However, a large number of asymptomatic individuals with a potential risk of developing a future CHD event remain undiagnosed in primary care settings [4].

In recent years, genome-wide association studies (GWASs) of CHD have also identified genetic risk factors. So far, over 150 single nucleotide polymorphisms (SNPs) have been identified to associate with CHD [5]. These include loci located in genes related to lipid metabolism, inflammation, transcriptional regulation, blood pressure, cell proliferation, neovascularization angiogenesis, NO-signalling, and vascular remodelling [5]. However, signals emerging from GWASs of CHD explain less than 10% of the heritability of CHD [6], prompting investigation into effects that may contribute to the ‘missing heritability’ in CHD [7].

Considering that CHD results in high mortality, it is crucial to develop accurate predictors for future CHD events, such as acute coronary syndrome, to enable early and effective interventions. At present, risk assessment of CHD events and other atherosclerosis-based cardiovascular events primarily relies on the use of different types of validated cardiovascular risk scores, for example the Framingham Risk Score, originally proposed over two decades ago [8]. Currently recommended risk scores include the SCORE risk prediction algorithms in Europe [9, 10], the QRISK risk prediction algorithms in the UK [11–13], and the pooled cohort equation atherosclerotic cardiovascular disease (ASCVD) risk score in the US [14]. These predict the risk of a 10-year first cardiovascular event, including CHD events. However, cardiovascular risk scores can also have limitations, for example, the ASCVD risk score can over- or under-estimate the risk of developing ASCVD in external samples [14–16]. These risk scores also do not consider further CHD risk factors, for example, genetic variants associated with the development of CVD.

Several studies have aimed to incorporate genotype information, often as a polygenic risk score (PRS) for CVD, into conventional CVD risk scores aiming to predict future or incident CHD (iCHD) events [17–20]. The majority of these integrated approaches do not report an improved discriminative capacity over use of conventional risk functions alone. However, most studies observe an improved reclassification of individuals in risk categories after PRS inclusion. At present, the clinical utility of integrating genetic information into conventional CVD risk prediction is under discussion. Current guidelines do not yet recommend the use of PRS in clinical practice to improve risk stratification [21].

These findings suggest that genetic variation may not significantly increase the performance of prediction in CHD, even though disease-associated genetic variants offer insights into the underlying biological mechanisms of disease. The relationship between genetic variation and disease may be mediated by epigenetic mechanisms. Epigenetic mechanisms are key regulators of gene function that can change in response to internal and external stimuli, including risk factors for CHD such as smoking [22]. Therefore, predictors of iCHD may benefit from considering additional layers of information, such as epigenetic variation.

Epigenetic mechanisms regulate gene expression and include DNA methylation, histone modification, RNA-associated silencing, and others. Among these, DNA methylation is the most studied epigenetic mark partly due to the development of multiple approaches to assay it including micro-arrays and bisulfite sequencing, as well as due to its relative stability allowing for profiling of previously collected stored DNA samples. Similar to GWAS, the association between phenotype and DNA methylation changes across the genome is assessed through epigenome-wide association studies (EWASs). Multiple EWASs have linked DNA methylation changes in certain 5′-cytosine-phosphate-guanine-3′ dinucleotides (CpGs) to CHD events. The majority of EWAS of CHD to date have considered retrospective CHD events at a single time-point, that is, in cross-sectional studies without longitudinal follow-up [23, 24]. Most studies have applied the Infinium HumanMethylation450 BeadChip (450k array) to explore blood DNA methylation variation at > 450,000 CpG-sites in primarily gene-centric regions across the human genome. However, unlike GWAS, CHD-associated DNA methylation changes may not be necessarily causal to CHD, but they may also be consequences of previous CHD events. To assess if epigenetic changes at individual CpG sites may help with the prediction of CHD, EWASs of iCHD and CHD risk factors are needed. Recently several EWAS of iCHD have been carried out, along with multiple EWAS of CHD risk factors. This narrative review explores DNA methylation signatures of iCHD, with a focus on EWAS findings from large-scale studies, to explore the potential of utilizing DNA methylation information for the risk assessment of iCHD, as well as to improve our understanding of how DNA methylation changes may contribute to iCHD.

## Epigenome-wide findings for iCHD

Several studies have assessed the association between iCHD and DNA methylation levels in the genome. Initial studies explored changes in global levels of DNA methylation in iCHD. Global DNA methylation levels can be assessed by using the methylation level of the repetitive long interspersed nuclear element-1 (*LINE-1*) as a proxy. Two studies have associated *LINE-1* hypomethylation in blood with iCHD [25, 26]. Global DNA methylation levels have also been explored by using the DNA methylation level of the ALU and Satellite 2 (AS) repetitive element as a proxy. In contrast to the hypomethylation findings with *LINE-1*, Kim et al. [27] found that AS methylation in peripheral blood leukocytes was higher (hypermethylated) in males with iCHD. Overall, the relationship between iCHD and global DNA methylation levels shows contradictory results, as discussed by Fernández-Sanlés and colleagues [28] in a systematic review. Therefore, further studies are needed at greater DNA methylation resolution to assess if individual CpG-site DNA methylation levels associate with iCHD.

Recently several large-scale EWASs identified differential methylation signatures at specific genomic regions to be related to the development of iCHD outcomes. In all cases whole blood DNA methylation levels were profiled in healthy individuals at baseline. After a longitudinal follow-up, a subset of participants subsequently developed an iCHD event (iCHD cases). Comparison of baseline levels of DNA methylation in iCHD cases and controls identified differential methylation signals in iCHD. In this section, we focus on locus-specific results from recent large-scale EWASs of iCHD outcomes and from large-scale EWASs of CHD risk factors with iCHD follow-up.

### EWASs of iCHD

Four recent large-scale EWASs have explored a range of iCHD outcomes. The most frequently studied outcome in EWASs of CHD is MI. An EWAS of incident MI was carried out by Guarrera et al. [26] using white blood cell DNA methylation profiles of 292 incident MI cases and 292 matched controls, with replication in 317 incident MI cases and 262 controls, both of European ancestry. The mean follow-up from baseline to iCHD outcome ranged between 5.64 (discovery cohort) to 6.9 years (replication cohort). The EWAS aggregated adjacent CpGs that have correlated methylation levels into 25,376 methylation regions, and identified one DNA methylation region consisting of 15 CpGs in the gene body of *ZBTB12* to be significantly hypomethylated in incident MI at a genome-wide false discovery rate (FDR) < 5%, after replication. The ZBTB12 protein has a potential function in transcriptional regulation, and a recent study associated *ZBTB12* gene body hypomethylation with faster blood coagulation triggered by tumour necrosis factor (TNF-α), as well as with increased white blood cell counts [29]. Guarrera et al. [26] then explored the relationship between *ZBTB12* DNA methylation levels and expression levels at *ZBTB12* and nearby genes in a separate sample set of approximately 80 healthy individuals, but no clear correlation was observed. The study also replicated previous findings of an iCHD association with *LINE-1* DNA methylation levels as markers of global methylation. The authors next incorporated DNA methylation levels of the *ZBTB12* 15 CpGs with *LINE-1* DNA methylation levels into a prediction model of MI based on age, sex, recruitment centre, smoking, body mass index (BMI), waist-to-hip ratio, lipid levels, blood pressure and menopausal status in women. Guarrera et al. found that by including DNA methylation in the model prediction of incident MI was improved in their replication cohort, as assessed by increased reclassification and discrimination (area under the receiver operating curves (AUC) improved from 0.66 to 0.69 for women and 0.7 for men) [26]. Overall, Guarrera et al.’s study discovered a novel blood DNA methylation signal for incident MI in *ZBTB12* and provided evidence for the value of incorporating DNA methylation in MI prediction [26].

Angina, coronary insufficiency, coronary revascularization and CHD death are also commonly used outcomes for CHD and are occasionally grouped along with MI as a phenotype of interest in CVD EWASs. Furthermore, the group of phenotypes occasionally also includes stroke, which is often caused by atherosclerosis. Several studies have explored the association between DNA methylation and the incidence of these CVD outcome groups. Westerman et al. [30] performed an EWAS associating iCHD (incident MI, angina, revascularisation, stroke and CHD death) with blood DNA methylation profiles in 2023 discovery subjects of multiple ancestries (1009 iCHD cases) and 2587 replication subjects of European ancestry (305 iCHD cases). Overall, 3 DNA methylation regions were identified and replicated for iCHD association at a genome-wide Bonferroni multiple testing correction, in the *SLC9A1*, *SLC1A5* and *TNRC6C* genes. Mendelian randomization analysis at 4 CpGs with methylation quantitative trait loci (meQTLs) in these regions identified one CpG (cg22304262) in *SLC1A5* with moderate evidence for a putative causal effect on iCHD. Furthermore, the *cis*-meQTL SNP for cg22304262 (rs8105903) can also alter the expression level of *SLC1A5* in blood in the GTEx (Genotype-Tissue Expression) data [31], suggesting that DNA methylation levels of cg22304262 may affect the expression of *SLC1A5. SLC1A5* encodes a sodium-dependent cellular amino acid transporter, and this gene was found to facilitate glutaminolysis [32]. Suppression of SLC1A5 was demonstrated to impair glutamine homeostasis in failing myocardium [33]. In summary, this study not only explored novel blood DNA methylation regions associated with iCHD, but also demonstrated a putative causal effect for one of the discovered signals [30].

In the largest EWAS of iCHD outcomes to date, Agha et al. [34] conducted an EWAS meta-analysis of iCHD events including coronary insufficiency, angina, MI, coronary revascularization and CHD death. The study explored 11,461 blood leukocyte DNA methylation profiles (1895 iCHD cases, including 1183 MI-only cases) from 9 population-based cohorts of multiple ancestries, with a mean follow-up of 11.2 years. Altogether, 52 differentially methylated CpG sites in blood were shown to be associated with iCHD (multiple outcomes) or MI-only at a genome-wide FDR < 5%. The iCHD-associated CpGs were annotated to genes involved in calcium regulation and kidney function. The study also performed Mendelian randomization at 10 of the CpGs that also had meQTLs, identifying two CpGs (cg26470101 near to *DLX2,* and cg07289306 neighbouring *MIR138-1*) with putative causal effects on iCHD. The authors also explored if the meQTLs for these two signals overlap published expression-QTLs (eQTLs) [31] and observed overlapping eQTLs for nearby genes (meQTLs for cg26470101 are also eQTLs for *ITGA6*; and meQTLs for cg07289306 are also eQTLs for lncRNA RP4-555D20.2), indicating that DNA methylation levels at these two CpG sites may also influence the expression levels of nearby genes. Interestingly, one of the 52 CpG signals (cg05820312 in *TRAPPC9*), was shown to have a putative causal effect on systolic blood pressure in a recent Mendelian randomization study [35], therefore independently validating its relevance to cardiovascular health. To sum up, the results from the largest epigenetic study of iCHD to date highlight novel signals and potential new mechanisms whereby DNA methylation changes related to calcium regulation and renal function may play a role in the development of CHD.

In the most recent EWAS of iCHD to date Navas-Acien et al. [36] assessed the association between blood DNA methylation levels and fatal or non-fatal iCHD in a discovery cohort of American Indian ancestry, with replication in four cohorts from multiple ancestries. As one of the largest EWAS of iCHD the study included 2321 individuals (748 iCHD cases) in the discovery sample with a mean follow-up from baseline to iCHD outcome of 19.1 years, and 7047 individuals (1145 iCHD cases) in the replication cohorts with a mean follow-up of 15.6 years across the replication samples combined. The authors applied both standard linear models and Elastic-Net models in the EWAS to account for the inter-associations among CpG sites. While the standard iCHD EWAS did not detect significant results, the Elastic-Net EWAS identified 505 differentially methylated positions (DMPs) associated with iCHD in the discovery sample, where 4 signals replicated in all replication cohorts, and a further 29 replicated in three out of four replication cohorts. Ten of these 33 replicated DMPs showed significant pooled hazard ratios (*p* < 0.05) in a Cox regression model, such that changes in methylation showed an overall consistent directionality with iCHD across cohorts. The 10 DMPs map to genes related to coronary artery lesions, cardiometabolic traits, blood pressure, pulmonary hypertension, stroke, lipid levels, and cell adhesion, indicating high relevance to iCHD. However, cohort-specific analyses also identified distinct iCHD signals in each sample, suggesting that iCHD methylome signals can also exhibit population-specific effects.

These four EWASs of iCHD have altogether identified 66 DNA methylation signals of iCHD after genome-wide correction for multiple testing (Table 1), although the majority of signals have small effect sizes. The signals are located in a wide range of genes, including genes involved in lipid metabolism, amino acid transport, ion transport, transcriptional regulation, RNA processing, immune system processes and tissue-specific regulations. Furthermore, causal inference analyses indicate that 3 CpGs (cg22304262, cg26470101 and cg07289306) may have putative causal effects on iCHD, as well as potential impacts on gene expression. However, the four EWASs of iCHD do not show overlapping DNA methylation signatures for iCHD, possibly due to population and phenotype heterogeneity, and small effects at the differentially methylated sites.

**Table 1:** Results from epigenome-wide association studies of incident CHD in blood (iCHD cases > 100)

Study	CHD outcome or phenotype	Sample size	Ancestry	Array type	CpGs or GRCh37/hg19 locations	Genes	Gene name
Guarrera et al. 2015 [26]	Incident MI	Discovery: 292 cases, 292 controls	European	Discovery: 450k array	Region consisting of 15 CpG sites (chr6: 31867698–31867919)	*ZBTB12*	Zinc finger and BTB domain containing 12
		Replication: 317 cases, 262 controls		Replication: MALDI-TOF mass spectrometry methylation assay			
Westerman et al. 2019 [30]	Incident MI, angina, revascularisation, stroke and coronary death	Discovery: 1009 cases, ≤ 1014 controls	Discovery: multiple ancestries	450k array	Region consisting of 3 CpG sites (chr1: 27440462–27440721)	*SLC9A1*	Solute carrier family 9 member A1
		Replication: 305 cases, ≤ 2282 controls	Replication: European				
					Region consisting of 6 CpG sites (chr19: 47287777–47288263)	*SLC1A5*	Solute carrier family 1 member 5
					Region consisting of 6 CpG sites (chr17: 76037034–76037562)	*TNRC6C*	Trinucleotide repeat containing adaptor 6C
Agha et al. 2019 [34]	Incident unstable angina, MI, coronary revascularization, coronary death	1895 cases, ≤ 9566 controls	Multiple ancestries	450k array	cg22617878	*ATP2B2*	ATPase plasma membrane Ca2+ transporting 2
					cg13827209	*TGFBR1*	Transforming growth factor beta receptor 1
					cg14185717	*BNC2*	Basonuclin 2
					cg10307345	*PTPN5*	Protein tyrosine phosphatase non-receptor type 5
					cg13822123	*PSME4*	Proteasome activator subunit 4
					cg23245316	*TSSC1*	Tumour suppressing subtransferable candidate 1
					cg24977276	*GTF2I*	General transcription factor IIi
					cg24447788	*PTBP1*	Polypyrimidine tract binding protein 1
					cg08422803	*ITGB2*	Integrin subunit beta 2
					cg01751802	*KANK2*	KN motif and ankyrin repeat domains 2
					cg02449373	*FUT1*	Fucosyltransferase 1
					cg02683350	*ADAMTS2*	ADAM metallopeptidase with thrombospondin type 1 motif 2
					cg05820312	*TRAPPC9*	Trafficking protein particle complex 9
					cg06639874	*MLPH*	Melanophilin
					cg06582394	*CASR*	Calcium sensing receptor
					cg02155262	*AGA*	Aspartylglucosaminidase
					cg12766383	*UBR4*	Ubiquitin protein ligase E3 component N-recognin 4
					cg05892484	*MAD1L1*	Mitotic arrest deficient 1 like 1
					cg03031868	*ESD*	Esterase D
					cg25497530	*PTPRN2*	Protein tyrosine phosphatase receptor type N2
					cg06596307	*IGF1R*	Insulin-like growth factor 1 receptor
					cg10702366	*FGGY*	FGGY carbohydrate kinase domain containing
					cg26470101	*DLX2*	Distal-less homeobox 2
					cg26042024	*ZFAT*	Zinc finger and AT-hook domain containing
					cg00466121	*ZNHIT6*	Zinc finger HIT-type containing 6
					cg04987302	*OTX2-AS1*	OTX2 antisense RNA 1
					cg08853494	*RCHY1; THAP6*	Ring finger And CHY zinc finger domain containing 1
							THAP domain containing 6
					cg26467725	*SLCO3A1*	Solute carrier organic anion transporter family member 3A1
					cg06442192	*ZNF541*	Zinc finger protein 541
					cg00393373	*ZNF518B*	Zinc finger protein 518B
					cg22871797	*CYFIP1*	Cytoplasmic FMR1 interacting protein 1
					cg18598861	*IRF9*	Interferon regulatory factor 9
					cg09777776	*ZNF254*	Zinc finger protein 254
					cg20545941	*MPPED1*	Metallophosphoesterase domain containing 1
					cg19935845	*TNXB*	Tenascin XB
					cg24423782	*MIR182*	MicroRNA 182
					cg19227382	*CDH23*	Cadherin related 23
					cg03467256	*HPCAL1*	Hippocalcin like 1
					cg25196881	*THBS1*	Thrombospondin 1
					cg02321112	*MNX1-AS1*	MNX1 antisense RNA 1
					cg00355799	*LOC339529*	/
					cg17556588	*PRRG4*	Proline-rich And Gla domain 4
					cg07289306	*MIR138-1*	MicroRNA 138-1
					cg22618720	*MIR5095*	MicroRNA 5095
					cg14010194	*GUCA1B*	Guanylate cyclase activator 1B
					cg24318598	*ANO1*	Anoctamin 1
					cg07015775	*ZNHIT6*	Zinc finger HIT-type containing 6
					cg21018156	*LINC01312*	Long intergenic non-protein coding RNA 1312
					cg07475527	*RCAN3*	RCAN family member 3
					cg20000562	*SFTA3*	Surfactant associated 3
					cg07436807	*STAMBPL1; ACTA2*	STAM binding protein like 1
							Actin alpha 2, smooth muscle
					cg14029912	*BHLHE40*	Basic helix-loop-helix family member E40
Navas-Acien et al. 2021 [36]	Incident fatal and nonfatal CHD include MI and coronary deaths	Discovery: 748 cases, ≤ 1573 controls Replication: 1145 cases, ≤ 5902 controls	Discovery: American Indian Replication: multiple ancestries	Discovery: EPIC array Replication: 450 k array	cg01620164	*FIGN*	Fidgetin, microtubule severing factor
					cg12479512	*RBSN*	Rabenosyn, RAB effector
					cg16604233	*COL11A2*	Collagen type XI alpha 2 chain
					cg07964553	*NEUROG2*	Neurogenin 2
					cg22293458	*VPS8*	VPS8 subunit of CORVET complex
					cg26955383	*CALHM1*	Calcium homeostasis modulator 1
					cg02628823	*MGAT4D*	MGAT4 family member D
					cg01297357	*ASIC1*	Acid sensing ion channel subunit 1
					cg09926486	*FRMD5*	FERM domain containing 5
					cg08622677	*PRMT8*	Protein arginine methyltransferase 8

### EWASs of CHD risk factors with iCHD follow-up

Multiple large-scale studies have carried out EWASs of CHD risk factors and subsequently explored how the resulting signals relate to iCHD outcomes. This approach may identify novel iCHD-associated CpGs beyond the signals identified from EWASs of iCHD alone, and help to understand how specific CHD risk factors contribute to biological mechanisms underlying iCHD development. Methylation signals could further contribute towards the development of iCHD methylation risk scores that together with traditional iCHD risk equations may improve the performance of iCHD risk prediction.

TNF-α is a pro-inflammation cytokine that plays an important role in atherosclerosis, which underlies most CHD events. A cross-sectional EWAS meta-analysis in 4794 subjects, with replication in a further 816 subjects of European ancestry, linked blood DNA methylation profiles to circulating TNF-α levels [37]. The results identified differential methylation at 2 CpG sites in *NLRC5* and 2 CpG sites in *DTX3L* and *PARP9*, where all three genes are involved in immune response. Upon exploring DNA methylation associations with gene expression *in cis* (*cis*-eQTMs), the authors found that all 4 CpGs were associated with expression of nearby genes in the discovery sample, but not in an external dataset. The authors then observed a significant association between DNA methylation levels of these 4 CpG sites and iCHD in 1895 iCHD cases (from Agha et al.’s study [34] with mean follow-up time of 11.2 years), which was a trans-ancestry population sample that overlapped with their discovery dataset. These results demonstrate that blood DNA methylation signatures of TNF-α may have biomarker potential for iCHD and towards methylation-based CHD risk prediction.

Lipids are important risk factors of CHD. Multiple EWASs have explored total cholesterol, LDL cholesterol and TG which are pro-atherogenic, and HDL cholesterol which is anti-atherogenic. Hedman et al. [38] performed an EWAS of lipids in whole blood samples from 2306 discovery subjects with replication in 2025 subjects of European ancestry. The study identified 33 associated CpGs after replication, of which one signal (cg27243685) in the *ABCG1* gene was also significantly associated with iCHD in 8-year (115 cases) and 10-year (78 cases) clinical follow-ups in their discovery sample. *ABCG1* encodes a cellular transporter protein that regulates lipid efflux, and *ABCG1* methylation levels have also been associated with retrospective CHD in recent candidate gene DNA methylation studies [39, 40]. Hedman et al.’s study also explored the association between DNA methylation at candidate CpG signals and gene expression in a subset of samples, and observed that methylation levels of cg27243685 negatively associated with *ABCG1* expression [38].

Obesity is another major risk factor of CHD [41]. Campanella et al. [42] carried out EWASs of obesity-related phenotypes (BMI, waist circumference, waist-hip and waist-height ratio) in peripheral blood leucocyte DNA methylation profiles from 1941 subjects with replication in 358 subjects from a European population, and where the peak methylation signals were then linked to future risk of MI (131 cases). Methylation levels at a CpG-site (cg12593793) in the *LMNA* gene identified from EWAS of waist-to-height ratio were inversely associated with risk of MI (follow-up time > 1 year) in the discovery sample. Polymorphisms and mutations in the *LMNA* gene are known to be related to abdominal adipocyte size [43], type 2 diabetes mellitus [43, 44] and cardiomyopathy [45]. The authors also assessed methylation associations with nearby gene expression levels, but no signals were found for cg12593793.

As previously discussed, risk scores are often used to aid the assessment of the likelihood of developing future CHD. Fernández-Sanlés et al. [46] developed an age-independent cardiovascular risk based on vascular age and multiple traditional cardiovascular risk factors including lipids, blood pressure, diabetes, smoking and obesity. They then investigated its association with genome-wide whole blood DNA methylation profiles in a discovery sample of 645 subjects, with replication in 2542 subjects of European ancestry. The results identified 8 CpGs in *ALPPL2, AHRR, PPIF, CPT1A, SBNO2* and in 3 intergenic regions. Using these 8 CpGs the authors developed a DNA methylation risk score (MRS) for predicting future CVD events. This MRS demonstrated an association with incident CVD (222 cases, median follow-up time = 7.66 years) in their replication cohort.

To conclude, to date 14 blood DNA methylation CpG signals have been identified from EWASs of CHD risk factors (including TNF-α, lipids, waist-to-height-ratio and age-independent cardiovascular risk) to be directly linked to the development of iCHD with a 7-to-12-year follow-up (Table 2). Altogether, these results identify genes and mechanisms of disease progression that may be targeted therapeutically. For example, the CpG sites map to genes involved in lipid metabolism, phosphatase activity and transcription activity, and one of these signals in *ABCG1* also associates with *ABCG1* expression. Although the CpG sites identified in these EWASs of CHD risk factors with iCHD follow-up do not overlap with the signals identified from EWASs of iCHD to date (described in the previous section) multiple signals were previously detected in EWASs of CHD risk factors without direct iCHD follow-up, and are outlined below.

**Table 2:** Results of EWASs of CHD risk factors in blood with incident CHD follow-up

Authors	Traits of interest in EWASs	Sample size	Ancestry	Array type	CpGs	Genes	Gene/RNA/protein full names
Aslibekyan et al. 2018 [37]	TNF-α	Discovery: 4794	Discovery: European	450k array	cg16411857	*NLRC5*	NLR family CARD domain containing 5
		Replication: 816	Replication: European		cg07839457		
		iCHD: 1895 cases, ≤ 9566 controls	iCHD follow-up: multiple ancestries		cg00959259	*DTX3L*; *PARP9*	Deltex E3 ubiquitin ligase 3L
					cg08122652		Poly(ADP-ribose) Polymerase family member 9
Hedman et al. 2017 [38]	TG, HDL-cholesterol	Discovery: 2306	European	450k array	cg27243685	*ABCG1*	ATP binding cassette subfamily G member 1
		Replication: 2025					
		iCHD: 193 cases, ≤ 2113 controls					
Campanella et al. 2018 [42]	Waist-to-height ratio	Discovery: 1941	European	450k array	﻿cg12593793	LMNA	Lamin A/C
		Replication: 358					
		iCHD: 131 cases, 195 controls					
Fernández-Sanlés et al. 2018 [46]	Age-independent cardiovascular risk	Discovery: 645	European	450k array	cg12547807	*LOC100506022*	Long intergenic non-protein coding RNA 02606
		Replication: 2542					
		iCVD: 222 cases, ≤ 1950 controls			cg27537125	*MIR4425*	MicroRNA 4425
					cg05951221	*AC068134.6; ALPPL2*	/
					cg21566642		Alkaline phosphatase, placental-like 2
					cg05575921	*AHRR*	Aryl-hydrocarbon receptor repressor
					cg19939077	*PPIF*	Peptidylprolyl isomerase F
					cg00574958	*CPT1A*	Carnitine palmitoyltransferase 1A
					cg18608055	*SBNO2*	Strawberry notch homolog 2

## The emerging blood-based DNA methylation signatures of iCHD

The signals detected from EWASs of iCHD and EWASs of CHD-risk factors with iCHD follow-up map to genes that are enriched for several molecular functions and biological processes. Enriched annotations include ion binding activity, transcriptional regulation, and regulation of cellular macromolecule biosynthetic process. Many of the identified signals related to iCHD have been previously identified as signals in EWASs of CHD risk factors in cross-sectional studies, without iCHD follow-ups. This section discusses candidate DNA methylation signals of iCHD that have emerged from recent EWASs and that have also previously shown associations in independent studies of specific iCHD risk factors (Table 3).

**Table 3:** Selected overlapping signals between EWASs of iCHD or with iCHD follow-ups, and EWASs of CVD risk factors and CVD-related traits

iCHD DNA methylation signals		CVD or CHD risk factors EWAS signals identified the same gene		
Study	Gene (CpG site, GRCh37/hg19 location)	Study	Phenotype/disease	Locus
Westerman et al. 2019 [30]	*SLC1A5* (Intragenic region consisting of 6 CpG sites located in chr19: 47287777–47288263)	Hedman et al. 2017 [38]	TG levels	cg02711608, 5′UTR/body
		Richard et al. 2017 [58]	Systolic and diastolic blood pressure	cg02711608, 5′UTR/body; cg22304262, 5′UTR/body
	*TNRC6C* (Intragenic region consisting of 6 CpG sites located in chr17: 76037034–760375623)	Abdulrahim et al. 2019 [52]	All-cause mortality	Intragenic region consisting of 6 CpG sites located at chr17: 76037035–76037563
Agha et al. 2019 [34]	*ITGB2* (cg08422803, TSS200/5′UTR)	Ligthart et al. 2016 [49]	CRP levels	cg18663307, 5′UTR/TSS1500
		Del Pilar Valencia-Morales et al. 2015 [62]	Atherosclerotic aorta	cg18012089, body
	*KANK2* (cg01751802, TSS1500)	Hedman et al. 2017 [38]	HDL-cholesterol levels	*cg01751802, TSS1500
		Zhang et al. 2017 [73]	Cardiac autonomic responses	cg26905268, TSS200
	*ADAMTS2* (cg02683350, body)	Gallego-Fabrega et al. 2016 [74]	Recurrence of ischemic stroke	cg09533145, body
	*ANO1* (cg24318598, 3′UTR/body)	Rask-Andersen et al. 2016 [23]	Cross-sectional MI	cg15269503, TSS200
Navas-Acien et al. 2021 [36]	*CALHM1* (cg26955383, TSS200)	Mendelson et al. 2017 [75]	BMI	*cg26955383, TSS200
Aslibekyan et al. 2018 [37]	*NLRC5* (cg07839457, TSS1500)	Ahsan et al. 2017 [76]	Levels of multiple protein biomarkers of inflammation and cancers	*cg07839457, TSS1500
		Hedman et al. 2017 [38]	Total cholesterol levels	*cg07839457, TSS1500
Hedman et al. 2017 [38]	*ABCG1* (﻿cg27243685, 5′UTR/body)	Pfeiffer et al. 2015 [54]	HDL-cholesterol levels; TG levels	cg06500161, body
		Demerath et al. 2015 [57]	BMI	cg06500161, body; *cg27243685, 5′UTR/body
		Campanella et al. 2018 [42]	BMI; waist circumference; waist-hip ratio; waist-height ratio	cg06500161, body
		Hidalgo et al. 2014 [55]	Insulin levels; HOMA-IR	cg06500161, body
Fernández-Sanlés et al. 2018 [46]	*ALPPL2* (cg05951221, intergenic region; cg21566642, intergenic region)	Abdulrahim et al. 2019 [52]	All-cause mortality	Intragenic region
		Zeilinger et al. 2013 [77]	Smoking	*cg21566642, intergenic region
		Fernández-Sanlés et al. 2021 [53]	Cross-sectional MI	*cg21566642, intergenic region
	*AHRR* (cg05575921, body)	Ligthart et al. 2016 [49]	CRP levels	*cg05575921, body
		Zhang et al. 2016 [48]	Smoking	*cg05575921, body
		Fernández-Sanlés et al. 2021 [53]	Cross-sectional MI	*cg05575921, body
		Portilla-Fernández et al. 2021 [50]	Carotid intima-media thickness	*cg05575921, body
	*CPT1A* (cg00574958, 5′UTR)	Hedman et al. 2017 [38]	TG levels	*cg00574958, 5′UTR; cg09737197, 5′UTR; cg17058475, 5′UTR
		Pfeiffer et al. 2015 [54]	TG levels	*cg00574958, 5′UTR
		Irvin et al. 2014 [56]	TG levels	*cg00574958, 5′UTR
		Demerath et al. 2015 [57]	BMI	*cg00574958, 5′UTR
		Richard et al. 2017 [58]	Systolic and diastolic blood pressure	*cg00574958, 5′UTR
	*SBNO2* (cg18608055, body)	Ligthart et al. 2016 [49]	CRP levels	*cg18608055, body
		Ek et al. 2016 [60]	GDF-15 levels	*cg18608055, body
		Kazmi et al. 2019 [78]	Hypertensive disorder of pregnancy	cg07573872, body

‘*’Denotes that the CpG site is the same as that identified in the EWAS of iCHD or with iCHD follow up

Several biomarkers of smoking have been identified in EWASs of iCHD or EWASs with iCHD follow-ups, including signals in the *AHRR* and *ALPPL2* genes. *AHRR* is a downstream target of the aryl hydrocarbon receptor (AHR) pathway, which facilitates the activation of enzymes and transporters involved in the elimination and biotransformation of toxins in the human body. *AHRR* helps mediate the transcription of AHR-dependent genes, functioning as a negative regulator in the AHR pathway [47]. DNA methylation of *AHRR* is the most consistent signature of tobacco smoking and has been shown to be a good biomarker for cardiovascular risk prediction [48], as well as being associated with serum CRP levels, a risk factor of CHD [49], and carotid intima-media thickness, an index of subclinical atherosclerosis [50]. *ALPPL2* is another consistently replicated smoking-methylation locus, where the protein is a membrane-binding glycosylated enzyme and is considered to be a highly specific tumour cell surface antigen [51]. DNA methylation of *ALPPL2* has also been associated with all-cause mortality [52]. Furthermore, a recent EWAS of CHD identified signals in *AHRR* (cg05575921) and near *ALPPL2* (cg21566642) to be associated with retrospective CHD with validation in independent iCHD samples [53].

CpG-sites in lipid metabolism genes have also been identified in EWASs of iCHD or EWASs with iCHD follow-ups, including in the *ABCG1* and *CPT1A* genes. *ABCG1* is a transporter protein regulating lipid efflux that plays an important role in preventing the accumulation of excessive cholesterol, thus the build-up of atherosclerosis in the human body. DNA methylation signals in *ABCG1* have previously been identified in a wide range of EWASs, including in EWASs of CHD risk factors such as HDL-cholesterol levels and TG levels [54], BMI, waist circumference, waist-hip and waist-height ratio [42], insulin levels and its assessment model HOMA-IR [55]. *CPT1A* encodes an important protein in lipid metabolism which mediates the transportation of long-chain fatty acid into the mitochondria. DNA methylation signals in *CPT1A* were previously reported in multiple EWASs of TG levels [38, 54, 56], BMI [57], and blood pressure [58].

Signals related to inflammation have also been identified in EWASs of iCHD or with iCHD follow up, including sites in the *SBNO2* and *ITGB2* genes. *SBNO2* encodes a transcriptional coregulator considered to be an inflammatory response factor in the central nervous system [59]. Differential methylation of the *SBNO2* gene was associated with serum CRP levels [49] and growth differentiation factor-15 (GDF-15) [60]. *ITGB2* encodes a component of the integrin pathway which is involved in the leukocyte adhesion process [61]. DNA methylation in the *ITGB2* gene has been associated with serum CRP levels [49], as well as variation in the atherosclerotic aorta [62].

Several EWASs of iCHD or with iCHD follow-up have carried out causal inference analyses of the peak signals. For example, DNA methylation levels in *SLC1A5* were shown to have a putative causal effect on iCHD [30]. *SLC1A5* encodes a sodium-dependent neutral amino acid transporter, which functions as a mediator of glutamine transport in cancer cell growth and survival [63]. CpG sites in *SLC1A5* were also reported to be associated with TG levels [38] and blood pressure [58] in independent EWASs. Apart from *SLC1A5*, Mendelian randomisation analysis also identified DNA methylation levels of 2 CpGs in the transcription activator *DLX2* and micro-RNA *MIR138-1*, respectively, which may have causal effects on iCHD. Although little is known about the exact links between these genes and CHD or CHD risk factors currently, these signals may be valuable for future research.

Altogether, EWAS of iCHD and EWAS of CHD risk factors with iCHD follow-ups have identified both novel DNA methylation signals, as well as signals with previously established relevance to cardiovascular health. These results give the opportunity for a better understanding of the development of CHD, as well as the potential for selecting new biomarkers of iCHD.

## Towards methylation-based iCHD risk prediction

Several large-scale EWASs have identified DNA methylation signals associated with iCHD, including both novel signals and changes previously identified to associate with CHD or CVD risk. Although the iCHD signals do not overlap across iCHD studies, multiple signals show promising biomarker potential (Table 3), and a small subset exhibit evidence for putative causal effects on iCHD. However, the potential of DNA methylation signatures discovered in EWASs of iCHD for the prediction of iCHD has not yet been fully assessed.

A few studies have explored the use of epigenetics towards the prediction of iCHD. Guarrera et al. [26] assessed the predictive value of incorporating the peak iCHD EWAS signals to predict iCHD within their replication dataset, showing improved discrimination and reclassification compared to the use of traditional risk factors alone. Other methylation predictors have also been developed in recent years using peak signals from EWASs of retrospective CHD. Fernández-Sanlés et al. [53] developed a methylation risk score for the prediction of CHD and CVD based on a two-stage EWAS of cross-sectional acute MI in peripheral blood in a European population sample and the association of EWAS peak signals with iCHD follow-up. However, they did not observe an increase in discrimination and reclassification when incorporating the methylation risk score into the Framingham risk function [8]. Given the limited overlap across signals from different EWAS of iCHD alone, these results suggest that methylation risk scores should also consider population-specific aspects, potentially reflecting different CHD risk profiles across populations.

In addition to methylation risk score predictors alone, multiple layers of -omic information have also been combined with DNA methylation signals to develop multi-omic risk score classifiers and predictors for iCHD. A series of studies were conducted to integrate genetic and epigenetic (DNA methylation) information using machine learning for the classification of retrospective CHD cases and controls [64], as well as for prediction of incident CHD [65, 66]. All studies showed improved sensitivity for classification or prediction of incident CHD compared to conventional risk models [8, 14]. The most recent study by Dogan et al. [66] developed an integrated genetic-epigenetic model for predicting 3-year iCHD from a training set consisting of subjects of European ancestry. The model, which incorporated data from three methylation loci and five SNPs, showed superior sensitivity compared to the Framingham risk model [8] and the ASCVD risk model [14] in an external validation set. In the external validation set, the sensitivity of prediction increased from 31% for the Framingham risk model and 69% for the ASCVD risk model, to 75% for the new combined genetic and epigenetic CHD model. These results show better performance of integrated genetic and epigenetic models compared to conventional clinical risk models for prediction of individuals at greater risk for future CHD. Another recent multi-omic approach by Palou-Márquez et al. [67] integrated DNA methylation and gene expression data using multi-omic factor analysis (MOFA). Four factors based on methylation variability were associated with CVD incidence, and two of these improved the prediction performance of the Framingham risk function [8] in integrated analysis. The findings indicate that methylation and multi-omic risk score predictors have potential to improve the performance of conventional clinical risk score predictors for predicting future iCHD. Moreover, replication of the approach in an independent cohort identified DNA methylation signatures at three genes that also contributed to a factor associated with MI in the replication sample. These results together with findings from EWAS of iCHD suggest an extent of population specificity in the DNA methylation signature of iCHD, which supports future explorations of population specific DNA methylation predictors of iCHD.

One difficulty in interpreting the iCHD results described so far is that alterations in different molecular mechanisms can give rise to separate CHD events that could then be grouped together into a single iCHD outcome in EWASs. For instance, the mechanisms underlying troponin-negative unstable anginas will likely differ from those behind troponin-positive acute coronary syndromes [68]. In the EWASs of iCHD described above, a few studies consider more than one subtype of iCHD event as the phenotype of interest, which introduces phenotype heterogeneity. For example, in some studies stroke may be used together with CHD events as a CVD outcome [30], although the underlying mechanisms may differ. To tackle this, larger studies should be undertaken with more homogeneous phenotypes, for example, restricting the outcome to a single type of CHD event. Ideally, clinical case ascertainment should be carried out where possible to classify disease cases in more details.

Further limitations to the iCHD EWAS studies discussed here relate to aspects of methylome profiling. For example, most of the studies measured blood DNA methylation profiles with the 450 k array in the discovery samples. The 450 k array only measures less than 2% out of 28.3 million CpG sites across the human genome. More recent methylome profiling microarray technologies, such as the ﻿Infinium MethylationEPIC BeadChip (EPIC array), double this genome coverage, but still only profile less than 4% of CpG sites genome-wide. Ultimately, whole-genome bisulfite sequencing efforts will be needed to assess the methylome signature of iCHD at full resolution. Additionally, the EWASs of iCHD described so far were conducted in blood. Blood methylation levels are biologically relevant to iCHD because, for one, blood leukocytes were demonstrated to be responsible for the inflammation reaction which starts the formation of atheroma [69]. Blood samples are also easy to collect and access, which makes it convenient to measure DNA methylation in large well-powered samples and use it as a prediction tool in clinical setups. However, atherosclerosis plaques are distributed in different arteries and display focality. Therefore, DNA methylation of whole blood may not be representative for the development of individual atheromas.

Finally, experimental follow-ups that explore the functional impact of methylation changes are often difficult to conduct following the discovery of EWAS signals. Most EWASs of iCHD or with iCHD follow-ups assessed whether EWAS signals may be related to corresponding changes in gene expression at nearby genes. Guarrera et al. [26], Hedman et al. [38], and Campanella et al. [42] compared DNA methylation profiles with gene expression profiles in internal or external samples, whilst Westerman et al. [30], Agha et al. [34] and Aslibekyan et al. [37] explored the relationship between meQTLs and eQTLs from publicly available databases. However, none of the iCHD EWASs so far have carried out direct experiments to confirm the biological function of altered methylation levels of the identified CpG signatures.

To conclude, the rapid development of epigenome-wide technologies has enabled research efforts that provide an opportunity to add an epigenetic layer into the prediction of iCHD. Multiple epigenetic signals have been identified in iCHD, but a large-scale meta-analysis across these EWASs has yet to be carried out. In the future, further exploration of larger cohorts, ideally with a more detailed and homogeneous classification for iCHD cases, is needed. In addition to blood, DNA methylation profiling of heart tissue or vascular walls, which has been very limited to date [70–72], would provide highly relevant findings. Additionally, more functional follow-ups need to be carried out to characterise the function of the identified iCHD DNA methylation signatures, as this may provide insights for the development of iCHD events and their prevention. Finally, studies that assess the predictive ability of DNA methylation signatures of iCHD should be performed in larger cohorts incorporating the identified DNA methylation signatures of iCHD. These efforts may also aid clinical interventions, for example, providing more accurate iCHD prediction that leads to informed decisions regarding the choice of intervention.

## Data Availability

Not applicable.

## Abbreviations

450k array: Infinium HumanMethylation450 BeadChip
ABCG1: ATP binding cassette subfamily G member 1
AHR: Aryl-hydrocarbon receptor
AHRR: Aryl-hydrocarbon receptor repressor
ALPPL2: Alkaline phosphatase, placental-like 2
AS: ALU and Satellite 2
ASCVD: Atherosclerotic cardiovascular disease
AUC: Area under the receiver operating curves
BMI: Body mass index
CHD: Coronary heart disease
*cis*-eQTMs: DNA methylation associations with gene expression *in cis*
CpG: 5′-Cytosine-phosphate-guanine-3′ dinucleotide
CPT1A: Carnitine palmitoyltransferase 1A
CRP: C-reactive protein
CVD: Cardiovascular disease
DLX2: Distal-less homeobox 2
DMPs: Differentially methylated positions
DNA: Deoxyribonucleic acid
DTX3L: Deltex E3 ubiquitin ligase 3L
EPIC array: Infinium MethylationEPIC BeadChip
EWAS: Epigenome-wide association study
FDR: False discovery rate
GDF-15: Growth differentiation factor-15
GTEx: Genotype-Tissue Expression
GWAS: Genome-wide association studies
HbA1c: Haemoglobin A1c
HDL: High-density lipoprotein
HOMA-IR: Homeostatic model assessment for insulin resistance
iCHD: Incident coronary heart disease
IDI: Integrated discrimination improvement
ITGA6: Integrin sub-unit α 6
ITGB2: Integrin subunit beta 2
LDL: Low-density lipoprotein
LINE-1: Long interspersed nuclear element-1
LMNA: Lamin A/C
meQTL: Methylation quantitative trait locus
MI: Myocardial infarction
MIR138-1: MicroRNA 138-1
MRS: Methylation risk score
NLRC5: NLR family CARD domain containing 5
NRI: Net reclassification improvement
PARP9: Poly (ADP-ribose) polymerase family member 9
PPIF: Peptidylprolyl isomerase F
PRS: Polygenic risk score
RNA: Ribonucleic acid
SBNO2: Strawberry notch homolog 2
SLC1A5: Solute carrier family 1 member 5
SLC9A1: Solute carrier family 9 member A1
SNP: Single nucleotide polymorphism
TG: Triglyceride
TNF-α: Tumour necrosis factor
TNRC6C: Trinucleotide repeat containing adaptor 6C
TRAPPC9: Trafficking protein particle complex 9
ZBTB12: Zinc finger and BTB domain containing 12
